# Association between body size and reservoir competence of mammals bearing *Borrelia burgdorferi* at an endemic site in the northeastern United States

**DOI:** 10.1186/s13071-015-0903-5

**Published:** 2015-05-30

**Authors:** Alan G. Barbour, Jonas Bunikis, Durland Fish, Klara Hanincová

**Affiliations:** Departments of Microbiology & Molecular Genetics and Medicine, University of California Irvine, 3012 Hewitt, Irvine, CA 92697-4028 USA; Department of Epidemiology and Public Health, Yale School of Medicine, New Haven, CT USA; Present Address: Department of Medicine, Vilnius University, Vilnius, LT-03101 Lithuania

**Keywords:** Tickborne, *Ixodes*, *Peromyscus*, Spirochete, Allometry, Lyme disease, Lyme borreliosis, *Borrelia miyamotoi*

## Abstract

**Background:**

The reservoirs for the Lyme disease agent, *Borrelia burgdorferi*, are dominated by several different small to medium sized mammals in eastern North America.

**Findings:**

To experimentally assess the competence of different mammalian species to transmit this pathogen to ticks, we carried out quantitative species-specific PCR of individual nymphal *Ixodes scapularis* ticks, which had been collected as replete larvae from animals captured at a field site in eastern Connecticut and then allowed to molt in the laboratory. The mammals, in order of increasing body mass, were the white-footed mouse, pine vole, eastern chipmunk, gray squirrel, Virginia opossum, striped skunk, and common raccoon. The prevalence of infection in the nymphs and the counts of spirochetes in infected ticks allometrically scaled with body mass with exponents of −0.28 and −0.29, respectively. By species, the captured animals from the site differed significantly in the mean counts of spirochetes in the ticks recovered from them, but these associations could not be distinguished from an effect of body size per se.

**Conclusions:**

These empirical findings as well as inferences from modeling suggest that small mammals on the basis of their sizes are more competent as reservoirs of *B. burgdorferi* in this environment than medium-to large-sized mammals.

## Findings

*Borrelia burgdorferi*, the predominant agent of Lyme disease in North America, is a generalist microparasite and exploits several different species of mammals and birds as reservoirs [1]. Transmission between vertebrate hosts in the eastern and central United States and adjoining areas of Canada is accomplished by the tick *Ixodes scapularis*. As a step towards further defining relative contributions of different hosts in *B. burgdorferi*’s natural cycle, we compared the competence of the white-footed mouse *Peromyscus leucopus* and selected other mammalian species in transmitting the pathogen to ticks. We used quantitative PCR to identify and measure the burden of *B. burgdorferi* in the nymphs derived from larvae that had naturally infested and fed on different hosts captured at a field site, Lake Gaillard, in Connecticut [2]. Reservoir competence was defined as the proportion of molted nymphs bearing *B. burgdorferi* after having fed as larvae upon a *B. burgdorferi*-infected animal. Quantitation of the spirochetes in each tick provided an additional assessment of competence. Nymphal ticks may be infected, as revealed by qualitative PCR or immunofluorescence, but they may be inadequate as vectors if spirochete counts are low [3].

Species-specific PCR for *B. burgdorferi* had been carried out on 1157 flat *I. scapularis* nymphs which had been recovered as engorged larvae from 62 mammals captured at the field site and then allowed to molt in the laboratory [4]. All trapping and handling procedures were approved by the Yale University Institutional Animal Care and Utilization Committee (Study Protocol 07596). Here we also report results for the relapsing fever agent *B. miyamotoi* [5]. The overall prevalences of *B. burgdorferi* and *B. miyamotoi* were 19.3 and 0.8 %, respectively, with a single (0.09 %) co-infection, the expectation for independence of infections [5]. These nymphal infection prevalences were similar to that reported for questing nymphs at this Connecticut site [6] and from other areas of the northeastern United States [5]. We carried out quantitation of *B. burgdorferi* in 641 ticks from 32 mammals, each with ≥4 recovered ticks, one or more of which was bearing ≥5 spirochete cells [5] (Table 1). The set comprised ticks from 3 eastern chipmunks (*Tamias striatus*), 14 white-footed mice (*P. leucopus*), 4 Virginia opossums (*Didelphis virginiana*), 3 common raccoons (*Procyon lotor*), 4 gray squirrels (*Sciurus carolinensis*), and 3 pine voles (*Microtus pinetorum*) [2], as well as one striped skunk (*Mephitis mephitis*). The methods for quantitative PCR and genotyping were as described [5, 7, 8]. The overall prevalence of *B. burgdorferi* in these examined ticks from infected mammals was 34 % (217 of 641), while infection prevalences in nymphs obtained from individual animals ranged from 4 to 100 %. The spirochete counts in 90 individual infected ticks from 14 mice approximated a log-normal distribution. The median count was 6833, the mean was 17,045, and the 5^th^ and 95^th^ percentile values were 600 and 37,099.

**Table 1:** B. burgdorferi burdens in ticks obtained as larvae from captured mammals and allowed to molt

Mammal ^a^	Number	Ticks examined	Nymphal infection prevalence	Mean spirochetes/infected tick (95% confidence interval) ^b^	Mean spirochetes/ examined tick ^c^
Chipmunk	1	60	0.23	2667 (1276–5572)	622
Chipmunk	2	14	0.36	15,922 (6637–38,194)	5686
Chipmunk	3	5	0.20	10,223	2047
Mouse	1	7	1.0	4446 (2523–7834)	4446
Mouse	2	4	0.75	10,990 (7096–17,022)	8243
Mouse	3	7	1.0	4943 (2512–9727)	4943
Mouse	4	7	0.86	714 (239–2138)	612
Mouse	5	7	1.0	7568 (3350–17,100)	7568
Mouse	6	7	1.0	6166 (4385–8670)	6166
Mouse	7	7	1.0	19,588 (11,324–33,884)	19,588
Mouse	8	7	1.0	9616 (5408–17,100)	9616
Mouse	9	7	1.0	7816 (5188–11,776)	7816
Mouse	10	5	0.80	2148 (863–5346)	1718
Mouse	11	7	1.0	25,468 (6668–97,275)	25,468
Mouse	12	7	1.0	4989 (3206–7762)	4989
Mouse	13	7	1.0	11,995 (9057–15,885)	11,995
Mouse	14	7	1.0	2188 (649–7379)	2188
Opossum	1	50	0.14	1786 (748–4266)	250
Opossum	2	27	0.59	755 (380–1500)	447
Opossum	3	50	0.04	1380 (540–3532)	55
Opossum	4	44	0.05	7 (6–9)	357
Raccoon	1	51	0.20	1358 (418–4416)	266
Raccoon	2	50	0.46	590 (289–1205)	271
Raccoon	3	49	0.08	2904 (1279–6592)	237
Skunk	1	11	0.09	3221	293
Squirrel	1	15	0.67	2301 (1042–5082)	1534
Squirrel	2	40	0.08	4753 (2661–8492)	357
Squirrel	3	4	0.50	2655 (625–11,272)	1327
Squirrel	4	8	0.75	946 (676–1324)	710
Vole	1	7	0.29	5970 (4721–7551)	1706
Vole	2	48	0.08	3750 (920–15,276)	312
Vole	3	15	1.0	15,346 (13,305–17,701)	15,346

^a^ Chipmunk (eastern chipmunk), mouse (white-footed mouse), opossum (Viriginia opossum), raccoon (common raccoon), skunk (striped skunk), squirrel (gray squirrel), and vole (pine vole)

^b^ Asymmetric confidence intervals from anti-logs of log-transformed counts

^c^ Product of prevalence (column 4) and mean spirochetes per infected tick (column 5)

The 8 different *B. burgdorferi* genotypes defined by the 16S–23S intergenic spacer (IGS) [7], and identified previously [2], did not appreciably differ in their counts in ticks recovered from *P. leucopus* and bearing a single genotype. The sample’s most frequent genotypes were IGS type 2 (*n* = 10), 3 (*n* = 8), and 5 (*n* = 5). The corresponding mean (95 % confidence interval) spirochete burdens by genotype were 5259 (2958–9352), 3612 (1234–10,571), and 5073 (2357–10,921) (ANOVA *F*_2,20_ = 0.26; *p* = 0.77). When all genotypes were grouped according to the RST typing scheme [9] as I (types 1 and 3; *n* = 11), II (type 2; *n* = 10, or III (types 4–8; *n* = 10), the spirochete burdens were 4158 (1804–9583), 5259 (2958–9352), and 8575 (4639–15,850), respectively (*F*_2,28_ = 1.05; *p* = 0.36).

We noted that species and individual animals represented in this survey varied not only in the prevalence of infections in their ticks but also in the counts of spirochetes in the ticks. For instance, for the majority of infected *P. leucopus* every tick recovered from an animal had *B. burgdorferi* and the spirochete burdens in these mouse-derived ticks were 3- to 9-fold higher than the counts in the ticks from opossums, raccoons, squirrels, and the skunk (Table 1). Voles and chipmunks resembled the mice in having high average counts per infected tick but, like the four larger mammals, tended to have lower prevalences of infections among the nymphs than was observed with the mice. The observations suggested an association with size of the mammal. We examined this possible relationship using the midpoint of the ranges of body masses in grams given by the Smithsonian National Museum of Natural History (http://www.mnh.si.edu/mna/main.cfm): vole (25.5), chipmunk (115), squirrel (544), opossum (2800), skunk (3250), and raccoon (6100). The value of 20 g for midpoint mass given for *P. leucopus* was same value as the mean of 298 adult *P. leucopus* captured at the field site in other collections (unpublished findings). These values closely corresponded with those provided by the AnAge database (http://genomics.senescence.info/species).

**Fig. 1:**
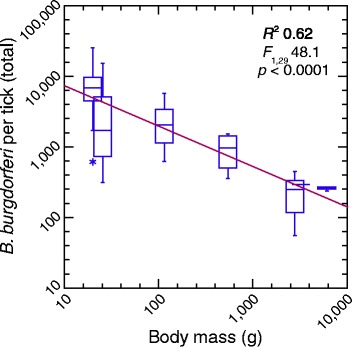
Box-whisker plots and regression of normalized *B. burgdorferi* cells per individual tick and body mass for seven Connecticut mammalian species from which larval ticks were collected after feeding and allowed to molt. Infection and bacterial counts were determined by quantitative PCR. In order of increasing size these were the white-footed mouse (*n* = 14), pine vole (*n* = 3), eastern chipmunk (*n* = 3), gray squirrel (*n* = 4), Virginia opossum (*n* = 4), striped skunk (*n* = 1), and common raccoon (*n* = 3). Each horizontal box indicates the first and third quartiles, and the indentation inside the box is the median. The 1.5× interquartile range is indicated by the horizontal line (whiskers) bisecting the box, and a value outside this range is indicated by an asterisk. The coefficient of determination (*R* ^2^), *F* statistic, and 2-tailed *p* value are shown

Nymphal infection prevalence (*i*_*P*_) decreased with body size (*w*): *i*_*P*_ = 1.8*w*^−0.29^; *R*^2^ = 0.43; *F*_1,29_ = 21.5; *p* <0.0001. Spirochete counts (*n*) in individual infected ticks similarly decreased with *w*: *n* = 15,284*w*^−0.28^; *R*^2^ = 0.37; *F*_1,29_ = 17.2; *p* <0.0001. The product of infection prevalence and mean spirochete load normalized the counts across every tick collected from an individual animal of each of the 6 species (Table 1). The log-transformed normalized counts differed by species (ANOVA; *R*^2^ = 0.66; *F*_6,24_ = 7.9; *p* <0.0001). But the species association for this estimate of reservoir competence was not distinguishable from an equally strong association with body mass alone (Fig. 1).

**Fig. 2:**
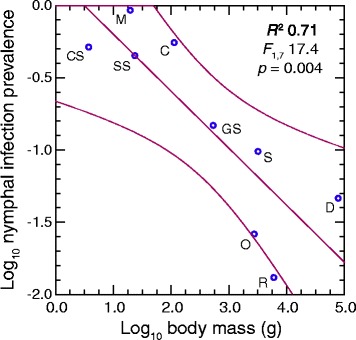
Log-log regression of mean nymphal infection prevalence on body mass in nine New York mammalian species from which larval ticks and were collected after feeding and allowed to molt. The data are from Table two of reference [10]. Infection was determined by immunofluorescence assay. The species were common shrew (CS), white-footed mouse (M), short-tailed shrew (SS), eastern chipmunk (C), gray squirrel (GS), Virginia opossum (O), striped skunk (S), common raccoon (R), and white-tailed deer (D). The 95 % confidence limits for the regression, the coefficient of determination (*R* ^2^), *F* statistic, and 2-tailed *p* value are shown

LoGiudice *et al.* performed a similar study of mammals and their attached larval ticks, which were collected in New York [10]. *B. burgdorferi* and *B. miyamotoi* were detected by genus-specific antibodies and immunofluorescence and not quantitatively, but the study assessed the prevalence of infection in all the ticks obtained from each animal. Analysis of the summarized data shows a similar negative association between body size over several orders of magnitude and a measure the authors called reservoir competence, which effectively was nymphal infection prevalence (Fig. 2). The species represented were, with exception of voles, the same as those examined here, with the addition of the common shrew (*Sorex cinereus*), short-tailed shrew (*Blarina brevicauda*), and white-tailed deer (*Odocoileus virginianus*). In an earlier study, which used the same criterion of reservoir competence, *P. leucopus* mice had comparatively greater competence than the larger chipmunk in an experimental study of this species and chipmunks [11].

We do not discount the roles of demography or life history, nor various species-specific resistances and susceptibilities to infection [4], in accounting for the observations reported here and by others. But appreciative of the heuristic tool of Occam’s Razor, i.e. among competing hypotheses, the one with the fewest assumptions should be selected, we propose consideration of an allometric explanation as well. One way in which body size may cash out in reservoir competence is in differing time periods for a vertebrate to achieve a state of infectiousness for ticks, namely, to reach a tissue or blood density above which feeding ticks would acquire the pathogen [1]. Noting that minimal infectious doses of *B. burgdorferi* appear to be 200 bacteria or fewer for dogs, laboratory rats, and mice [12–14], we posit that inocula required for acquiring this pathogen do not appreciably scale with body size of the host. With a discrete deterministic model we estimated the time (*t*) in days it took for a pathogen to reach a particular density (*c*) per gram of body mass when infections began with one organism. For this exercise, body size, *w*, was in grams and the pathogen’s doubling rate, *v*, was in hours.

$$ t=\frac{1.44\left( \ln w+ \ln c\right)}{v} $$

**Fig. 3:**
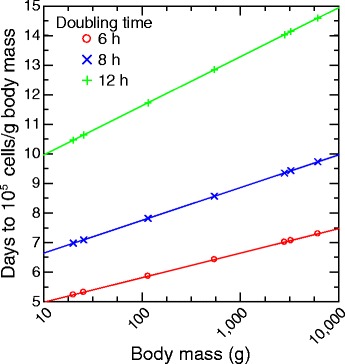
Discrete deterministic model of time in days to reach 10^5^ cells per gram (g) of body mass from start of infection of animals of different masses and for different in vivo growth rates (doubling time) for a bacterial pathogen that equally infectious for all tissues. See text for the description of the model. The body masses correspond to the masses of the species presented in Fig. 1

Figure 3 shows the association between body size and time to infectiousness in this simple model for growth rates that ranged from a 6 h doubling time typical of *in vitro* cultivation of *B. burgdorferi* [15] to 1 of 12 h, which may more accurately represent proliferation in a naïve vertebrate host [16]. The target density was 10^5^ spirochetes per gram of tissue, which was the median density of spirochetes in ear tissue in naturally-infected white-footed mice [5]. The body sizes were those for species of this study. Between the smallest and largest animals there was a difference of 1.5 to 4 days before the putative threshold for infectiousness in this model was reached, depending on the *in vivo* growth rate.

In a dynamical model of epidemics of De Leo and Dobson, transmission scaled allometrically with body size, increasing with size when transmission was density-dependent and decreasing with size for frequency-dependent transmission, which more suitably applies to vector-borne infections [17]. The empirically-determined scaling exponents of −0.29 for prevalence of infection and −0.28 for bacterial counts per infected tick were close to the body size exponent of −0.26 for the account of frequency-dependent transmission in De Leo and Dobson’s model. The importance of body size of either the host or the macroparasite on the epidemiology of an infection was also explored by Morand and Poulin [18] and by Bolzoni *et al.* [19]. Taken together with these other perspectives on the phenomenon, our findings prompt further study of the effect of body size on reservoir competence. A priority is on examining this relationship with other vector-borne pathogens and their natural hosts.
